# Multiblock Elastomers TPEAA and TPEEA: Physical Structure and Properties

**DOI:** 10.3390/ma14247720

**Published:** 2021-12-14

**Authors:** Joanna Rokicka, Katarzyna Wilpiszewska, Jolanta Janik, Beata Schmidt, Anton Nikiforov, Svetoslav Volfson

**Affiliations:** 1 Department of Organic Chemical Technology and Polymer Materials, Faculty of Chemical Technology and Engineering, West Pomeranian University of Technology, Piastów Ave. 42, 71-065 Szczecin, Poland; katarzyna.wilipszewska@zut.edu.pl (K.W.); jolanta.janik@zut.edu.pl (J.J.); beata.schmidt@zut.edu.pl (B.S.); 2 Department of Chemistry and Technology of Elastomer Processing, Faculty of Rubber and Elastomer Technology and Processing, Kazan National Research Technological University, 68 Karl Marx Street, 420015 Kazan, Russia; antonnikifor@gmail.com (A.N.); svolfson@kstu.ru (S.V.)

**Keywords:** poly(ester-ether-amide), poly(ester-aliphatic-amide), multiblock terpolymers, elastomers, phase structure

## Abstract

A three series of terpolymers composed of the blocks PTMO (M_PTMO_ = 1000 g/mol) or DLAol (M_DLAol_ = 540 g/mol), PA12 (M_PA12_ = 2000 g/mol) and xGT (DP_xGT_ = 2) with various chemical compositions of ester block were obtained. The series differ in the chemical structure of the flexible block and weight content of the soft phase. The effect of the number of carbons dividing the terephthalate groups on the synthesis, structure and properties of these elastomers has been investigated. To confirm assumed chemical structure Carbon-13 (^13^C NMR) and Proton (^1^H NMR) Nuclear Magnetic Resonance and Fourier-transform Infrared Spectroscopy (FT-IR) were used. The influence of chemical compositions of ester block on the thermal properties and the phase separation of obtained systems were defined by Differential Scanning Calorimetry (DSC), Dynamic Mechanical Thermal Analysis (DMTA) and Wide Angle X-ray Scattering (WAXS). The mechanical and elastic properties were evaluated.

## 1. Introduction

Thermoplastic elastomers (TPEs) are materials that combine the flexibility of traditional elastomers with the ease of processing characteristic for thermoplastics [1,2,3,4]. Most TPEs are essentially phase-separated systems. Due to phase separation, TPE has no less than two phases: soft and hard [1,5,6,7]. The soft phase (responsible for elastic properties) should have a relatively low modulus of elasticity, glass transition temperature (Tg) and density, and the hard phase (responsible for the strength and processing properties) must have a relatively high elastic modulus, glass transition temperature (Tg) or melting point (Tm) and density. The hard phase represents the thermally reversible physical cross-links. Strong cohesion of block building in the hard phase has a stabilizing effect on the phase structure of the entire polymer system [1,2,3,8]. The properties of TPE are the result of combining the individual characteristics of the blocks; therefore, the key to the design of these materials is to select the system components in such a way that a stable micro- or nanostructure is formed. Currently, the main soft blocks for TPEs are PEG [9,10,11], PTMO [12,13,14,15,16], and polyetheramine [17,18], enabling good flexibility and distribution of the rigid block domains. The hard phase consists mainly of amide blocks, PA6 [19], PA11 [20,21], and PA12 [22,23], which ensure strong interactions inside the domains and high mechanical strength. The functional and processing properties of TPE depend, among others, on their physical structure and the key factors affecting it, such as the content of individual blocks and their chemical structure; molecular weight, as well as mutual miscibility, is necessary to design new polymers dedicated to strictly defined purposes. The presented literature from recent years comprehensively describes two-block systems. Their nanostructure and its influence on the properties of copolymers are well known, and further research focuses mainly on the design of new materials [24,25,26,27,28,29].

However, there is still relatively few papers on ternary systems. The inclusion of a third component in the diblock copolymer macromolecule significantly affects the physical structure of the polymer [30,31,32,33,34,35,36,37,38]. Depending on the type, molecular weight, and proportion, the third block introduced may be:

- insoluble in the other two—new phase structures will probably arise;
- partially soluble in one and insoluble in the other—it may change the properties of the phase in which it partially dissolves and may possibly form new phase structures;
- completely soluble in one and insoluble in the other—changes the properties of the phase in which it dissolves;
- partially soluble in the other two—it may enlarge the transition area and may change the properties of the phases in which it partially dissolves;
- completely soluble in one and partially soluble in the other—changes the properties of the phase in which it dissolves, probably affects the properties of the entire system; or
- completely soluble in the other two—changes the properties of both phases, can destroy the heterophasic structure [39].

Due to the complexity of ternary systems, a detailed study of intersegmental, intermolecular, and interfacial interactions is critical to understanding the nature of these materials. Therefore, this paper describes the synthesis of new ternary TPEs and analyzes their physical structure and phase composition.

## 2. Materials and Methods

### 2.1. Materials

Synthesis of terpolymers were carried out using the commercially available substrates, namely: dimethyl terephthalate (DMT)—Chemical Plant “Elana”, ethylene glycol (2G), 1,3-propanediol (3G), 1,4-butanediol (4G), 1,5-penatnediol (5G), 1,6-hexanediol (6G)—Sigma-Aldrich, poly(oxytetramethylene)diol with molecular weight 1000 g/mole (PTMO)—Du Pont, linoleic alcohol dimer (DLAol)—Croda, a titanate catalyst (TiO_2_/SiO_2_)—Sachtleben Chemic GmgH, thermal stabilizer (Irganox 1010)—Ciba Geigy, caprolactam, dodecano-12-lactam, sebacic acid (SA), adipic acid (AA)—Aldrich Chemie. The lactam and the dicarboxylic acid are the substrates prepared in our laboratory: α,ω-dicarboxylic oligo(laurolactam) (PA12) with the number average molecular weight 2000 g/mole.

### 2.2. Determination

The limiting viscosity number [η] of the terpolymers in phenol-tetrachloroethylene mixture (60:40 vol/vol) was determined by an Ubbelohde viscometer II at 30 °C. Optical melting points T_m_ were determined using a Boethius microscope (HMK type Franz Kustner Nacht KG) at a heating rate of 2 °C/min. Blurring of sample edges set initial melting temperature. Complete transformation to an eye drop marked the final melting point. Hardness (H) measurements were performed on a Shore A and D apparatus (Zwick, type 3100) according to PN-80/C-04238. Swelling in the benzene and water (pH2O, pb) were performed according to PN-66/C-08932.

Fourier-transform Infrared Spectroscopy (FT-IR) analyses were carried out on a Nexus spectrometer. Spectra were acquired in the scan range 4000–530 cm^−1^, with a resolution of 4 cm^−1^ and a total of four scans. Carbon-13 (^13^C NMR) and Proton (^1^H NMR) Nuclear Magnetic Resonance were performed on a Bruker Avance spectrometer at 75 and 400 MHz, respectively.

Breaking force, the Young module, elongation at break, and elastic recovery were collected at room temperature with an Instron 5982. These parameters were measured per each sample on ten replicates. Speed of moving clamp was 100 mm/min. Mechanical hysteresis loops of two types were received. The first loop was obtained by stretching terpolymers at constant elongation to 100%. After, elongation specimens were allowed to relax and return to the initial gauge length. The cycle was repeated five times. Based on this measurement, the percent of elastic recovery and permanent deformation of specimens were calculated. The second was received by stretching a polymer from 10% to 100% at elongation growing by 10%. Before testing, all specimens were conditioned without any stress in the standard atmosphere (22 ± 2 °C, 65 ± 5% humidity) for at least 24 h.

Wide Angle X-ray Scattering (WAXS) analyzes were performed using a Geiger Flex diffractometer with a wide angle goniometer. Copper CuKα radiation with a wavelength of 1.54 Å monochromatized with nickel was used. Diffraction was recorded in the range 2θ angles from 5 to 38 with a goniometer speed of 2 °C/min at room temperature. Differential Scanning Calorimetry (DSC) tests were carried out using a Q100 TA Instrument. Hermetic aluminum pans were used to seal TPE samples weights in the range of 7–9 mg. Measurements were carried out in the heating–cooling–heating cycle in the temperature range −90–250 °C with a heating ramp 10 °C/min. The value of the glass transition temperature is given at the onset point and the variation of heat capacity ΔCp is calculated at the Tg midpoint. Dynamic Mechanical Thermal Analysis (DMTA) were performed using a MK II Polymer Laboratories in the temperature range from −90 °C to the melting point of the sample, with a heating rate of 1 °C/min.

### 2.3. Sample Preparation

Samples were obtained by injection moulding on BOY 35A. The process was carried out at 50 MPa at a temperature approximately 20 °C higher than the melting point determined with a Boethius microscope. Discs for WAXS analysis were obtained on hydraulic press at 10 MPa and a temperature approximately 10 °C higher than the melting point.

### 2.4. Synthesis of TPEs

**Figure 1:**
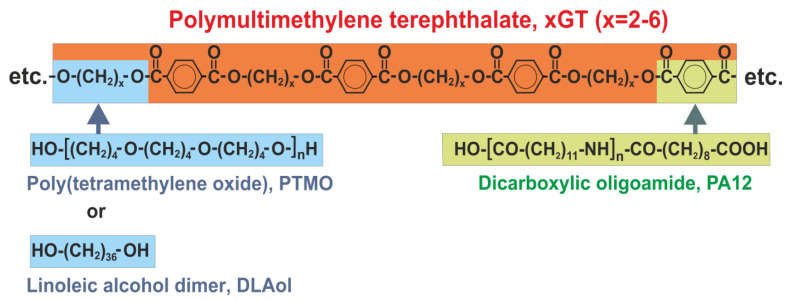
Polymultimethylene terephthalate modification.

The copolymers were obtained by chemical modification of the poly(multimethylene terephthalate) macromolecule. A certain part of the fragments originating from the terephthalic acid was substituted by the dicarboxylic oligoamide block, and some fragments derived from glycol were replaced by the oligoetherdiol or aliphatic block. The scheme of modification is presented in Figure 1.

**Figure 2:**
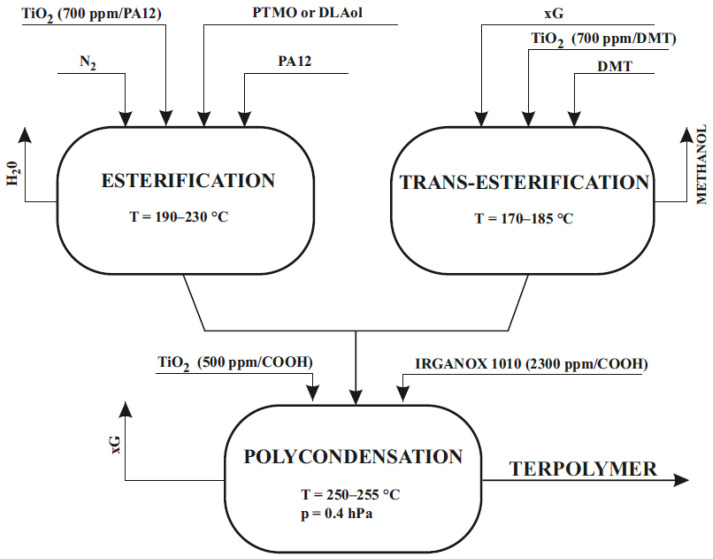
Synthesis of thermoplastic elastomers (TPEs).

Copolyesters were prepared by the typical melt polycondensation method. The synthesis consisted of two steps with the use of the catalyst. The first stage was the trans-esterification reaction of dimethyl terephthalate (DMT: 2 moles) with glycol (xG: 3 moles) leading to the formation of polyester and the release of methanol (GTGTG + 4CH_3_OH↑) and the esterification (which was taking place simultaneously in the different reactor) of α,ω-dicarboxylic oligo(laurolactam) (PA12) with oligo(oxytetramethylene)diol (PTMO) or linoleic alcohol dimer (DLAol) (by-product is water). The obtained product is PTMO-b-PA12-b-PTMO + 2H_2_O↑. From the respective amounts of methanol and water, it was concluded that the conversion ratio in the trans-esterification reaction was 95% and the degree of esterification was 90% (degrees was expressed as the weight ratios of the released methanol or water to the respective stoichiometric amounts of these products). Condensation polymerization of the obtained mixed intermediates was the second step in the preparation process (GTGTG + PTMO-b-PA12-b-PTMO → [PTMO-b-PA12-b-PTMO-b-(GT)x]n + G↑. The titanium catalyst was used in all stages of the synthesis. During the polycondensation, thermal and antioxidant stabilizers were used. Synthesis conditions are presented in Figure 2.

The molecular weight of PTMO should not exceed 1400 g/mole—this provides a good separation of the soft and hard phase and the composition of the intermediate phase is suitable (the comparable content of blocks PA12 and PTMO and a certain number of PTT blocks stabilizing the structure of this phase). The molar ratio of PTMO to PA12 should be in the range of 2 and 3.5—the interphase has the optimal ester-ether-amide structure and degree of polycondensation should contain in the range of 2–5—this determines the appropriate degree of separation of the hard and soft phase and does not cause formation of the second crystallizing (ester) phase.

## 3. Results

A three series of terpolymers composed of the blocks PTMO (M_PTMO_ = 1000 g/mol) or DLAol (M_DLAol_ = 540 g/mol), PA12 (M_PA12_ = 2000 g/mol) and xGT (DP_xGT_ = 2), with various chemical compositions of ester block, were obtained. The series differ in the chemical structure of the flexible block and the weight content of the soft phase. In the series I and II, the molar ratio of blocks was constant. Due to the significant difference in molar weight of flexible blocks, series I and III copolymers differ significantly in the content of flexible segments. In order to examine the effect of the weight content of flexible blocks, a third series of materials in which the weight content of flexible blocks similar to the series I was synthesized. This arrangement facilitated a detailed investigation of the influence of the chemical structure and the content of the soft phase on the properties of the obtained materials.

The type, the molar ratio, and the weight ratios of reagents used for the synthesis are presented in Table 1.

### 3.1. Chemical Structure

The assumed chemical structure of synthesized terpolymers has been confirmed by FT-IR, ^1^H NMR, and ^13^C NMR methods.

**Figure 3:**
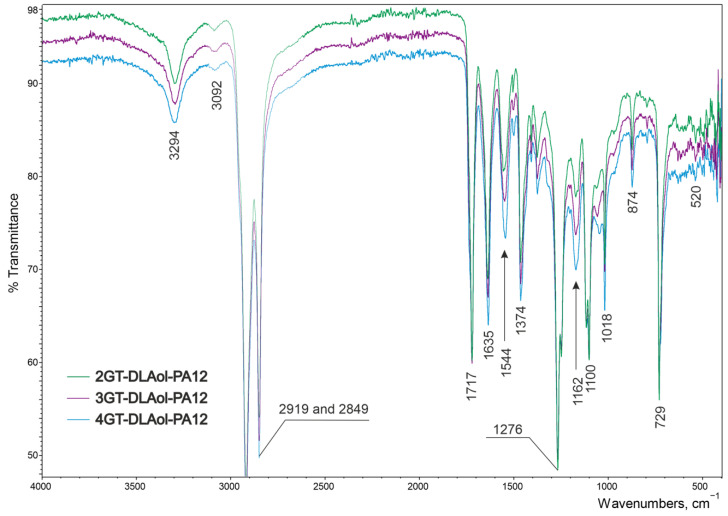
FT-IR spectra of terpolymer xGT-DLAol-PA12 where x = 2, 3 and 4.

**Figure 4:**
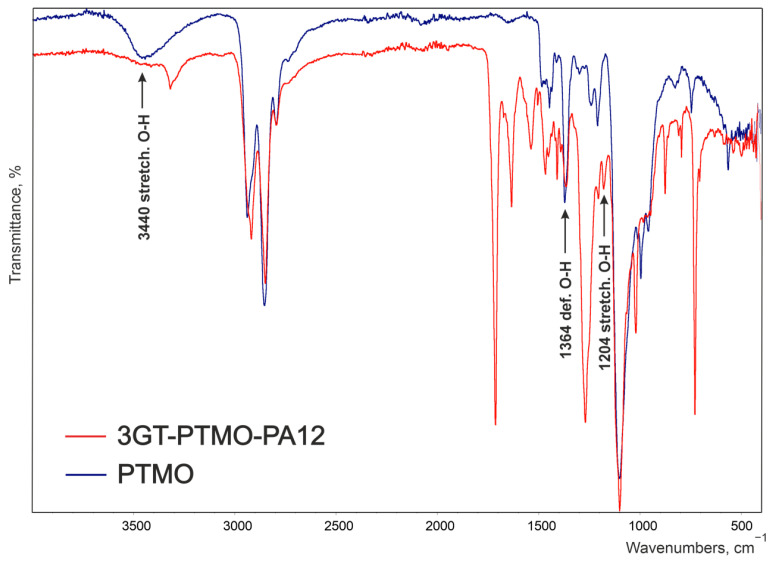
FT-IR spectrum of the reference terpolymer (3GT-PTMO-PA12) and PTMO homopolymer.

**Figure 5:**
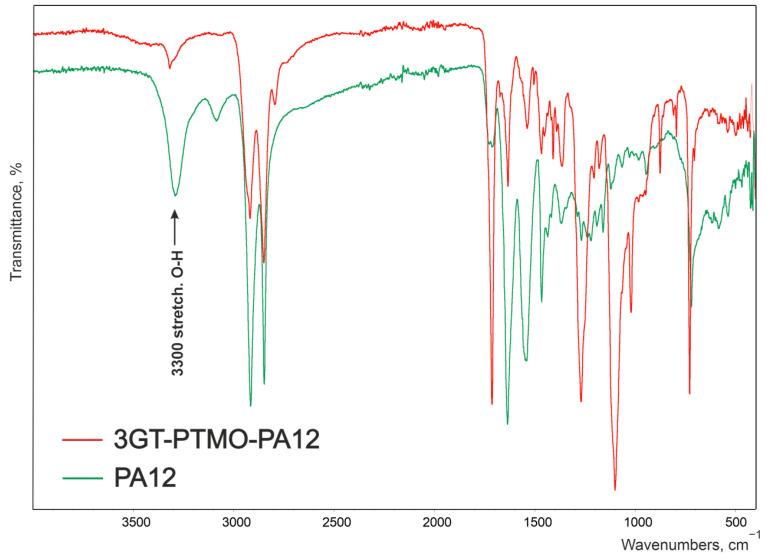
FT-IR spectrum of the reference terpolymer (3GT-PTMO-PA12) and PA12 homopolymer.

Obtained copolymers all had characteristic FT-IR bands for esters, aliphatic diols, or ethers and amides, which are presented in Table 2 and in Figure 3, Figure 4 and Figure 5.

There were no significant differences in the spectrograms of individual materials within the same series. The effect of extending the aliphatic chain derived from glycol is imperceptible during FT-IR analysis (Figure 3).

Figure 4 and Figure 5 shows the spectrogram of the obtained ester-b-ether-b-amide (3GT-PTMO-PA12) and PTMO and PA12 homopolymer. There is no large broad adsorption band at the 3440 cm^−1^ wavenumber on the terpolymer spectrum (Figure 4). A significant reduction in signal intensity at 1364 and 1204 cm^−1^ can also be observed. The vibrations of the hydroxyl end groups of the polyether are responsible for these bands. The absence of these signals or their significant weakening indicates a decrease in the concentration of end groups and thus the incorporation of the ether block into the terpolymer macromolecule.

The signal at the wavenumber of approximately 3300 cm^−1^, corresponding to the stretching vibrations of the O-H groups of the end carboxylic groups in the terpolymer, is much weaker than on the spectrum of the PA12 homopolymer (Figure 5). This proves the incorporation of the amide block into the terpolymer macromolecule.

**Figure 6:**
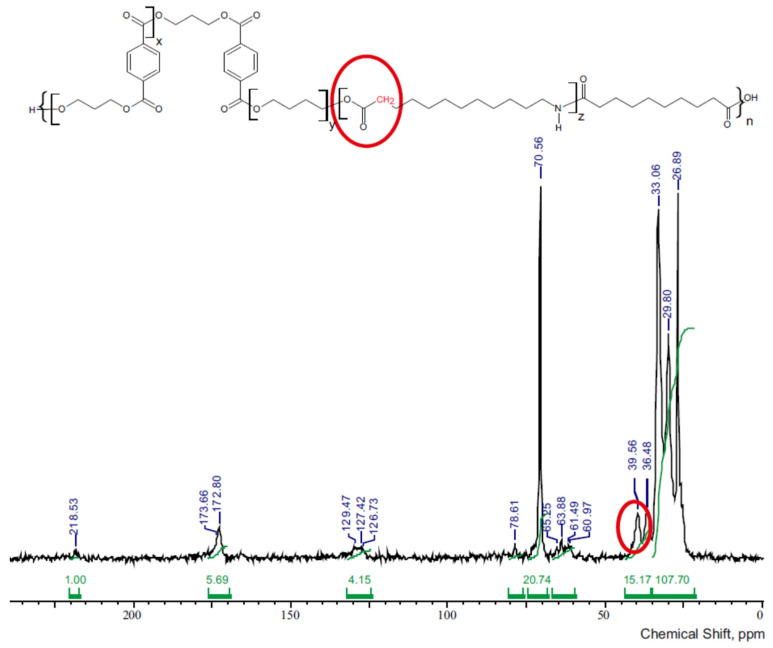
^13^C nuclear magnetic resonance (NMR) spectra of terpolymer 3GT-PTMO-PA12.

Figure 6 shows ^13^C NMR spectra of multiblock terpoly(ester-ether-amide). There are all characteristic groups found in esters, ethers, and amides. The signals were noted in the range of 26–218 ppm and their detailed interpretation can be found in Table 3.

There are no signals originating from the carbon atoms of the carboxyl group in the ^13^C NMR spectra (Figure 6). The 39.5 ppm chemical shift signal corresponds to an aliphatic–aliphatic ester group. Such a bond can only be formed by reaction of the amide carboxyl end groups with an ester or ether block. Considering the above, it can be concluded that amide was incorporated into the macromolecule of the polymer.

**Figure 7:**
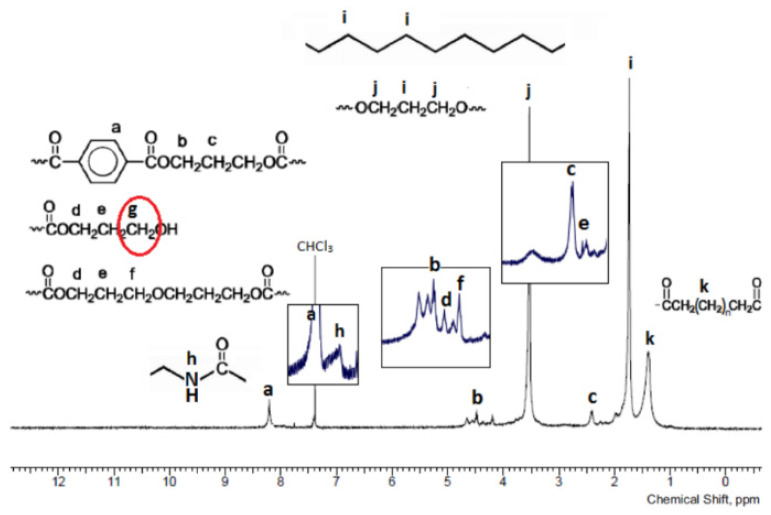
^1^H NMR spectra of terpolymer 3GT-PTMO-PA12.

Figure 7 shows the ^1^H NMR spectrum of the reference terpolymer (3GT-PTMO-PA12) and its interpretation in relation to the chemical structure of the material.

The absence of intense signals from the protons of the carboxyl (in the range of 10–13 ppm) and hydroxyl group (the characteristic slim singlet in the 1–5 ppm range) suggests the incorporation of both the amide and ether block in the terpolymer macromolecule. It is important that groups containing acidic protons (such as -OH or -NHCO-) tend to replace their protons with solvent-derived deuterium. As a result, signals from these groups can be significantly blurred or even completely disappear. However, the absence of free hydroxyl groups is confirmed by the lack of signals from the protons of the methylene group connected to the hydroxyl group (designated as ‘g’ in Figure 7).

### 3.2. Main Properties of TPEs

The selected properties of TPEs were presented in Table 4.

The limiting viscosity number values above 1.25 dl/g ensure the satisfactory molecular weights and good mechanical properties of the TPEs. The [η] values of obtained polymers have proved that they are composed of the macromolecules with satisfactory large molecular weights.

Swelling is the consequence of interaction between a solvent and a polymer matrix. It expresses the affinity and exchange of enthalpy between the two phases, is the first step before total solvation occurs (if possible), and it causes an increase in 3D geometry. The swelling behavior of a polymer depends on the nature of the polymer, polymer-solvent compatibility and degree of cross-linking, and also allows determination of the quality and quantity of the amorphous phase for partially crosslinked systems. Swelling is a tool to measure the crosslinking density, and for linear polymers, swelling will reflect how good/bad the solvent is for the polymer. When an uncrosslinked, amorphous, glassy polymer phase is in contact with a thermodynamically compatible solvent, the solvent will diffuse into the polymer. The absorption capacity depends on the mobility of the polymer chains and the cohesive interactions between them. Results gained from the water swelling test indicates that all the polymers obtained are hydrophobic; since the swelling does not exceed 4%, this indicates that water penetrates the continuous phase of the polymer very slightly.

In each series, as the distance between the terephthalic groups increased, the absorption capacity of benzene increased, also causing softening of the material. Flexible blocks create polymer matrix with a decreased degree of crystallinity. The chemical structure of the flexible block does not affect the absorbability of benzene. Comparing the I and III series, where the soft phase content is similar, it is seen that the swelling reaches the same value.

### 3.3. Physical Structure

Samples of the homopolymer and terpolymer were heated, cooled, and reheated at temperatures between −90 °C to 250 °C. In the temperature range of −90 °C < T < 25 °C, changes in the soft phase can be observed. On the other hand, above 25 °C, the curve characterizes the thermal features of the hard phase. The glass transition temperature Tg, the specific heat capacity ΔCp, the crystallization temperature Tc, the enthalpy of crystallization ΔHc, the melting temperature Tm, and the enthalpy of melting ΔTm, were determined based on the thermograms.

The proper assessment of the physical structure of obtained terpolymers was preceded by a detailed DSC and WAXS analysis of homopolymers and copolymers constructed from a combination of terpolymer building blocks (Table 5). For each homopolymer, the Hildebrandt solubility parameters were calculated (Table 6) to estimate their mutual solubility. The obtained δ values were compared with the literature data. The values of the theoretically calculated parameters coincide with the empirically determined values available in the literature. This allows conclusion regarding the correctness of the enumeration method used, and the results obtained can be used for further analyzes.

The glass transition temperature of PTMO, in the low temperature region, is Tg_1_ = −90 °C (determined in our laboratory; the literature states −89–91 °C). The glass transition temperature of copolymers containing this block (samples 6 and 7 in Table 5) is, respectively, Tg_1_ = −77 °C and −67 °C. The difference between the glass transition temperature of the homopolymer and copolymer is 13 °C for poly(ester-amides) (PEA) and 23 °C for poly (ester-ethers) (PEE). It is assumed that stiffening the ends of the flexible block chain with the chemical bond with the rigid block increases the glass transition temperature by about 5–10 °C and the interactions between each (soft and hard) phases may increase this temperature by a further 5 °C. It can therefore be assumed with high probability that Tg of the PEA corresponds to Tg of pure PTMO. This indicates that PA12 blocks with a molecular weight of 2000 g/mol do not dissolve or slightly dissolve in the PTMO block phase. This conclusion is also confirmed by the analysis of solubility parameters. The square of difference in the solubility parameters for PA12 and PTMO blocks is 22.6 MPa (several times higher than the miscibility value Δδ^2^ ≤ 3 MPa), thus indicating their mutual incompatibility. The increase in Tg by more than 20 °C cannot be explained by the stiffening of the chain ends and interphase interactions. Probably, the dissolution of ester blocks in the ether block phase is responsible for the increase in Tg PEE. However, the analysis of the solubility parameter values δ of these blocks indicates that they are mutually insoluble (Δδ^2^ = 5.9 MPa). For each polymer mixture the critical molecular mass of the components, below which, despite the thermodynamic incompatibility of components, the system will create a homogeneous mixture can be determined. For the PTT-PTMO system, the critical molecular weight of the ester block is 600 g/mol, therefore PTT blocks with a mass less than 600 g/mol will be mixed with the PTMO fraction. Due to the specific nature of PEE synthesis in the reaction environment, short ester sequences persist for a long time, which can be successfully blocked on both sides by PTMO segments. These short, non-crystallizing ester fragments dissolve in the amorphous phase of PTMO blocks and increase its Tg. Further analysis of the low temperature region shows that, in PEA, the PTMO blocks that build the amorphous phase retain their ability to crystallize. However, the same blocks do not crystallize in PEE. It confirms the conclusions about the partial dissolution of the ester sequences in the PTMO block phase.

In the high-temperature region (above room temperature), endotherms corresponding to the melting of the crystalline phase can be observed. It is worth noting that in the case of the copolymer PA12-PTT, there is a phenomenon of lowering the temperature below the melting temperatures of both components, the so-called depression of melting points. That indicates a significant defect in the crystal structure of the system. The reduction of Tm relative to the homopolymer can also be observed with the PTT-PTMO copolymer (approximately 30 °C). Most probably, this is due to the previously indicated mutual miscibility of PTT and PTMO blocks. The Tm of the copolymer PA12-PTMO is equal to Tm of the PA12 homopolymer, which again confirms the insolubility of these blocks.

**Figure 8:**
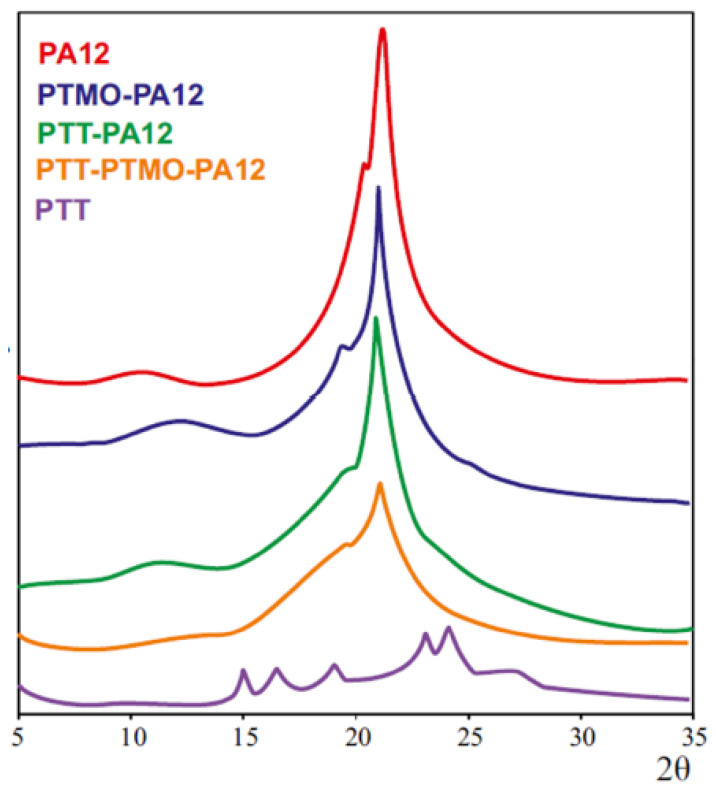
Wide Angle X-ray (WAXS) diffractograms of homo- and copolymers.

To qualitatively examine the crystal structure of homopolymers and terpolymers, the DSC analysis was supplemented with WAXS tests. The results are shown in Figure 8.

For the PTT homopolymer, six extreme peaks with the values of 2Θ reflections 15.08°, 16.51°, 19.17°, 23.19°, 24.39° and 26.91° can be observed. The diffractogram of the PA12 homopolymer has two obvious diffraction peaks: a weak diffraction peak at 10.14° and the sharp main peak at 21.75° with a side peak at 19.75°, which are the result of overlapping reflections from two polymorphic structures, γ and α, of PA12. In the diffraction patterns of terpolymers, there are only reflections with reflection angles 2Θ corresponding to the angle values of the PA12. It can therefore be concluded that in the multiblock copolymers with rigid PTT and PA12 blocks, only PA12 blocks are responsible for forming the crystalline phase. A decrease in the intensity of the reflections of terpolymers compared to the homopolymer PA12 and copolymers PTMO-PA12 and PTT-PA12 indicates the specific physical structure of the terpolymer PTT-PTMO-PA12. The obtained terpolymers are complicated systems whose properties depend on, among others, the mutual miscibility of the blocks. Most probably, the PTT block partly mixed with both the PTMO and PA12 phases significantly affects the properties of these phases, increases the volume of the interphase, and is the compatibilizer of the whole system. Short ester sequences penetrating between the crystalline PA12 blocks can be a spatial barrier during the crystallization process. It results in a significant disorder on the crystal structure of terpolymers.

**Figure 9:**
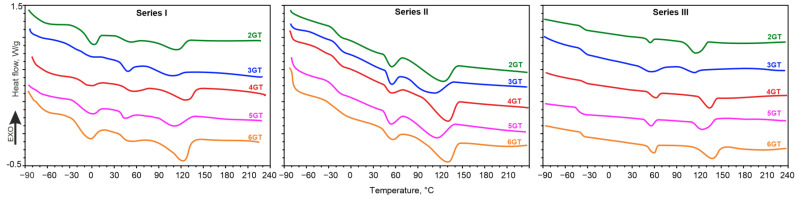
Differential Scanning Calorimetry (DSC) second heating curves of the obtained terpolymers.

The effect of the chemical structure of ester blocks on the phase structure have been determined by the DSC method. The results are presented in Figure 9 and Table A1, Table A2 and Table A3 in the Appendix A.

Characteristic for all series is the constant glass transition temperature of the low temperature region and the equal span of the glass transition interval. Since Tg,_PTMO_ was −90 °C and Tg of the amorphous phase of terpolymers (series I) was around −70 °C, it can be concluded that the soft amorphous phase constituting the matrix is composed of PTMO blocks and a small amount of short ester sequences dissolved therein. For series II and III, similar conclusions can be drawn. However, the increase in Tg of terpolymers containing the DLAol block is much larger than for terpolymers containing PTMO (30 °C for II and 40 °C for III series). It can be concluded that ester blocks dissolve much better in the phase of aliphatic blocks than in the phase of ether blocks. The solubility of ester blocks in the phase of the flexible blocks is essentially influenced by their chemical structure. The 10 °C difference in Tg between the II and III series results only from the increase in the amorphous phase content. The change of glycol in ester blocks does not affect the quality and degree of disturbance of the amorphous phase. With an increase of the number of methylene groups separating terephthalate groups in the ester block, the melting point of the crystalline fraction of PTMO blocks (series I) slightly increases. This may indicate that the amorphous fraction of ester blocks slightly increases the amorphous matrix of synthesized polymers.

In the high temperature region, there are two melting endotherms. The thermal effect at about 50 °C is most likely due to the melting of small, defected xGT crystallites. The second endotherm determines the temperature and enthalpy of melting of the crystalline phase of the terpolymers. The crystal melting endotherms reach their maximum in the range of 108–133 °C. Melting peaks are fuzzy with broad bases. The enthalpy value along with the shape of the endotherm proves the size and perfection of the crystalline phase. The more the melting temperature is close to that of the pure PA12 homopolymer (169 °C), the crystalline phase of terpolymer is more organized. In all series, the endotherm determining the melting temperature of the crystalline hard phase slightly shifts as the number of methylene groups in the ester block increases, its maximum moves towards higher temperatures and the enthalpy of melting increases. This indicates the better phase separation of xGT and PA12 blocks. These conclusions are also confirmed by the analysis of the solubility parameters of these blocks. The increase in the number of methylene groups in the ester block causes an increasing thermodynamic incompatibility of the mixture of ester and amide blocks (the square of the difference between Hildebrand’s solubility parameters rises from 3.7 MPa for 2GT/PA12, up to 10.4 MPa for the 6GT/PA12 block mixture).

**Figure 10:**
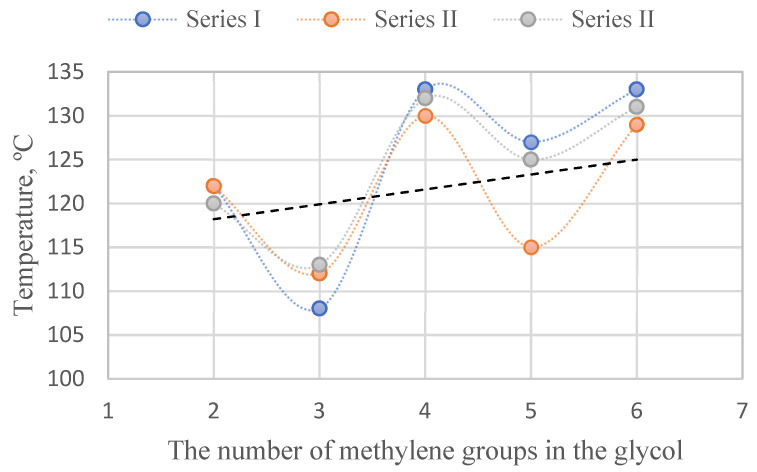
The parity rule of melting point of the terpolymers.

For each of the terpolymers, the phenomenon of depression of the crystalline hard phase’s melting temperature can be observed. This is the result of the dissolution of short ester sequences in PA12 and deterioration of the quality of the crystalline phase, and may also indicate the plasticizing effect of xGT blocks on the hard phase. In addition, a parity rule can also be observed for the whole series (higher melting temperatures of terpolymers with an even number of methylene groups in the glycol from the melting temperature values of both adjacent materials with an odd number of these groups) (Figure 10). Polyesters of even numbered diols exhibit higher melting temperatures than those of odd numbered glycols [48,49]. The occurrence of the odd-even effect at the melting point of the copolymers may suggest that the TPEs crystalline phase is composed of a mixture of PA12 and xGT blocks.

It is worth noting that terpolymers with 3GT ester blocks (polymers 2, 7 and 12) are characterized by the smallest heat melting values and their melting endotherms are broad and oblate. This indicates a relatively weak phase separation and greater miscibility of ester blocks with the phase of PA12 blocks. It can therefore be expected that in these materials the interphase will be the thickest.

**Figure 11:**
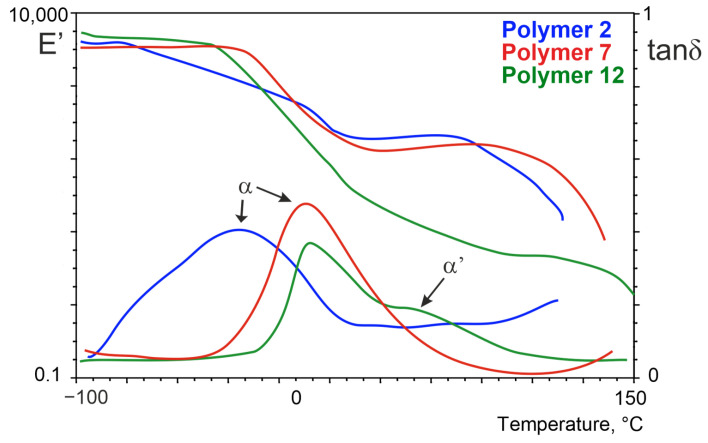
Dynamic Mechanical Thermal Analysis (DMTA) for the terpolymers with 3GT ester block; molar ratio: PTMO/PA12 = 3 (polymer 2), DLAol/PA12 = 3 (polymer 7), DLAol/PA12 = 5.5 (polymer 12).

The relaxation behavior of all samples was studied by DMTA, measuring the storage modulus (E′), the loss modulus (E″), and the loss tangent (tanδ). The effect of the temperature on the dynamic mechanical properties of TPE depending on the degree of polymerization of ester block is presented in Figure 11.

The obtained temperature spectra are typical for thermoplastic elastomers. The spectra of the storage modulus have three temperature regions, in which the courses E′ = t(T) differ significantly. In the temperature region from −100 °C to −70 °C (Series I), −20 °C (Series II) and −30 °C (Series III), the obtained TPEEAs exhibited a constant value of storage modulus above 1 GPa characteristic for the glass state. In the next region, viscoelasticity relaxation processes appear and decrease of modulus was observed. A further increase of temperature caused the occurrence of a wide “plateau” of elastic state, which, above a temperature of 120 °C, terminates by a rapid lowering at the point of crystallite melting of the hard block phase. All terpolymers exhibit broad maxima of damping, and their shape is the result of overlapping of several relaxation transitions relevant to melting, and the glass transition of the amorphous phase and interphase (block mixture occurring at the interface). The shape of these maxima is influenced by dispersion in the amorphous phase of non-crystallizable ester and amide blocks. The xGT-PTMO-PA12 tanδ curves have a broader relaxation peak in contrast to xGT-DLAol-PA12 terpolymers, which have one narrow and high damping peak. Probably, in these terpolymers the interphase is smaller because of good mixing of ester blocks with DLAol matrix (more short ester sequences are in the amorphous phase than in the interphase). Maximum α’ peaks can be observed in terpolymers of series III. This is a result of the relaxation effect of the amorphous phase of PA12 (high content of aliphatic amorphous blocks and their poor mixability with amide blocks causes better phase separation). Within each series, there are no significant changes in the DMTA curves.

**Figure 12:**
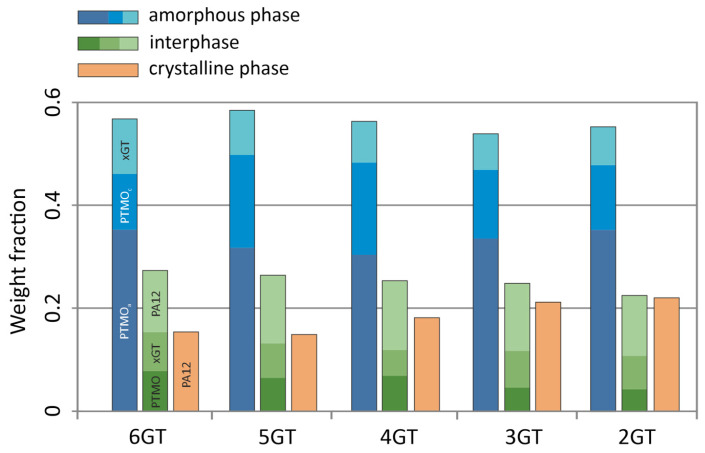
The phase compositions of the terpolymers of series I.

**Figure 13:**
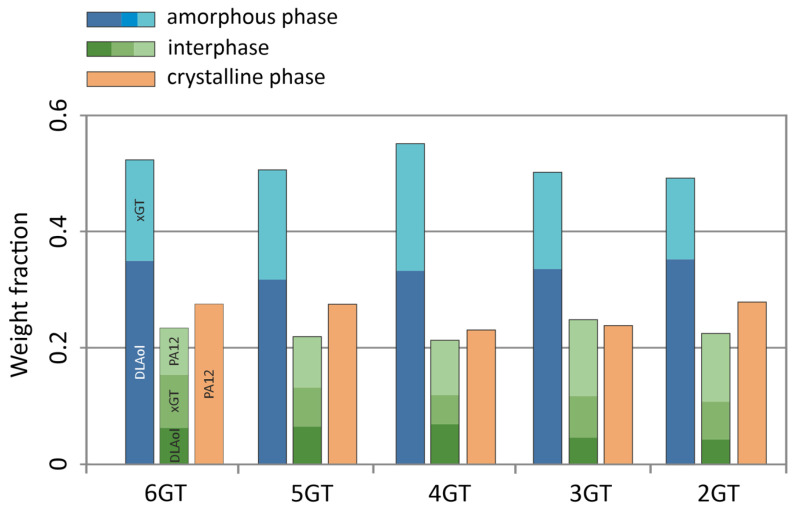
The phase compositions of the terpolymers of series II.

**Figure 14:**
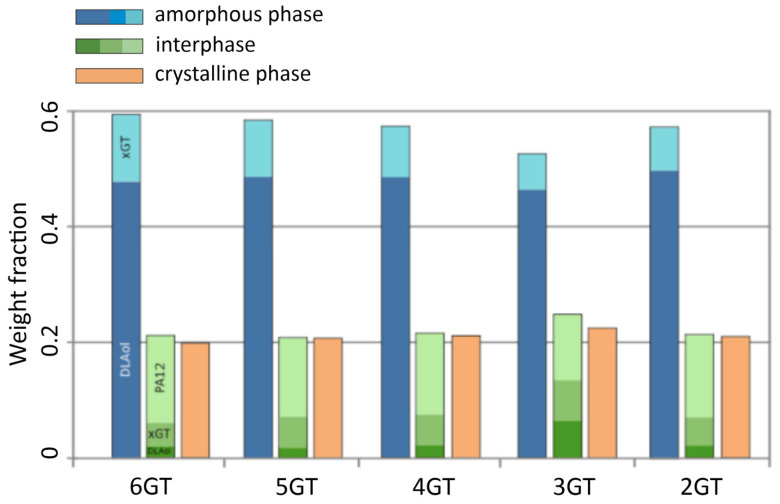
The phase compositions of the terpolymers of series III.

The DSC and DMTA analysis allowed determination of the quantitative phase composition of obtained terpoly(ester-b-ether-b-amides) and terpoly(ester-b-aliphatic-b-amides), which were presented in Figure 12, Figure 13 and Figure 14. The phase composition is calculated from the following data:

- Degree of phase separation (SRs) of the soft phase (Equation (1)):

SRs = [(ΔCp_exp_/ΔCp_a_) + (ΔHm_exp_/ΔHm_c_)]·w_s_^−1^ (1)

where ΔCp_exp_ is the experimental heat capacity of the terpolymer, ΔCp_a_ is the heat capacity of the amorphous homopolymer, ΔHm_exp_ is the enthalpy of melting of the terpolymer, ΔHm_c_ is the enthalpy of melting of the crystalline homopolymer, and w_s_ is the weight fraction of the soft phase (real solution of xGT in PTMO or DLAol) in the polymer (SRs + w_x,xGT_)

- Degree of crystallinity (w_c_) of the hard phase (Equation (2)):

w_c_ = (ΔHm_exp_/ΔHm_c_)·w_h_^−1^ (2)

where w_h_ is the weight fraction on the crystallizing hard block of the polymer.

With the following assumptions—the PA12 blocks are insoluble in the amorphous phase, the PTMO(DLAol)/xGT fraction can be treated as a separate system, the glass transition temperature of the flexible block, due to the immobilization of the chain-ends by chemical bonds, is higher by 10 °C than that determined for homopolymer, and the xGT blocks do not crystallize in the presence of PA12—the fraction of short xGT segments dissolved in amorphous phase (w_x,xGT_) was calculated from the Gordon–Taylor Equation (3). This enables the calculation of the weight fraction of the PTMO(DLAol)/xGT real solution and the degree of soft phase separation of the terpolymer.

w_x,xGT_ = w_a_(Tg_a_ − Tg_exp_)/(k(Tg_exp_ − Tg_xGT_) (3)

where w_a_ is the weight fraction of flexible block (PTMO or DLAol) and xGT in the amorphous phase, Tg_exp_ is the glass transition of the terpolymer, Tg_a_ is the glass transition of homopolymer PTMO or DLAol, Tg_xGT_ is the glass transition temperature of xGT block, and k is a coefficient of intermolecular interactions, which reflects the magnitude of deviation from the additive change of Tg as a function of the solution composition.

The obtained TPEs of series I comprises the elastomers, in which the fraction of the flexible block PTMO is prevailing. Along with an increase of the number of carbons separating the terephthalate groups in the ester block in TPEEA, the fraction of the hard phases slightly decreases, whereas the content of the interphase is growing. The interphase composition is constant. A characteristic for this series is a constant, large fraction of the PA12 blocks of the interphase, amounting about 12 wt.%/polymer, and a constant fraction of the xGT block in the soft phase.

The soft phase of the terpolymers of series II consists of a mixture of DLAol blocks with an amorphous fraction of ester blocks (this was also confirmed by the analysis of solubility parameters of xGT and DLAol blocks). The content of xGT blocks in the amorphous phase is about 30 wt.%. Due to the above, only a small part of the DLAol blocks goes to the interphase, which in practice has an ester-amide structure. The content of individual phases is relatively constant and does not change linearly with the number of methylene groups in the ester block.

In the III series, the content of the amorphous phase reaching 60 wt.%/polymer is constant. The amorphous phase is composed of aliphatic blocks contaminated with a small amount of ester sequences. The interphase has an ester-amide structure with a significant majority of PA12 blocks. The type of glycol used during the synthesis does not significantly change the phase composition of these materials; however, the content of ester blocks in the phase of DLAol blocks slightly increases. This is related to the increasing amorphous fraction of xGT blocks (longer carbon chain).

### 3.4. Mechanical Properties

**Figure 15:**
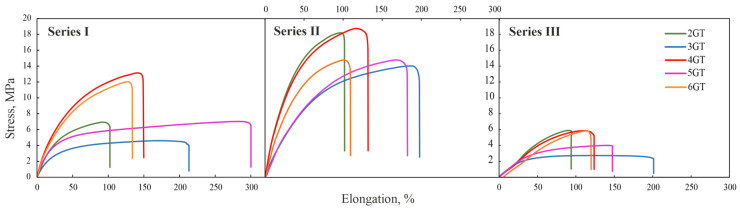
Strain–stress curves of TPEs.

The mechanical properties were evaluated using a static stretching technique. Tensile strength (σ), elongation at break (ε) and Young’s modulus (E) were determined and presented in Table 7. Figure 15 shows the dependence strain-stress of the obtained terpolymers. The strength curves of terpolymers are typical for elastomers for which no yield point and bending upwards (occurring during induced crystallization) exists.

**Figure 16:**
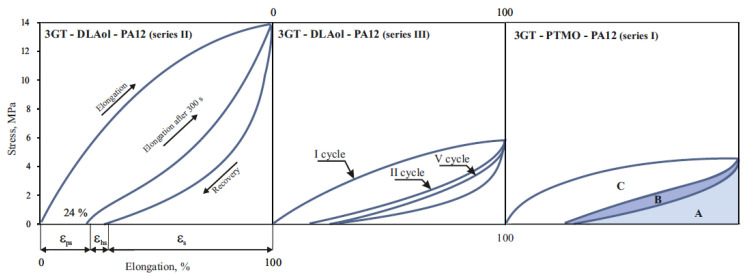
Mechanical hysteresis loops at a constant elongation of 100% of terpoly(ester-aliphatic-amide) of series II and terpoly(ester-ether-amide) of series I, where the ester block was 5GT: ε_s_—elastic elongation; ε_hs_—delayed high-elastic elongation; ε_ps_—permanent elongation; A—area proportional to the elastic energy dispersed; B—area proportional to the high-elastic energy dispersed; C—area proportional to the energy accumulated.

**Figure 17:**
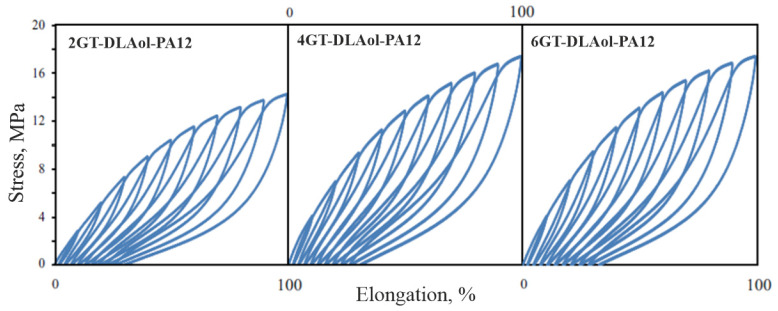
Mechanical hysteresis loops with elongation growing by 10% of the terpoly(ester-aliphatic-amide) of series II.

To evaluate the elastic properties of TPE, two types of mechanical hysteresis loops characterizing elasticity and stress relaxation in selected polymers were made. The first type was obtained by extending the samples five times by 100% and then releasing the tension (Figure 16); the second type was obtained by stretching the samples from 10% to 100%, with a 10% increase in extension (Figure 17).

Relaxed terpolymer’s flexible blocks have the form of a tangled bundle. During stretching, the sample deforms and the macromolecules stretch. After releasing the tension, the sample returns to the previous shape, and the macromolecules to previous configurations. If during the stretching of the sample the macromolecules have been stretched with a simultaneous change of position, then permanent deformation of the material takes place. The ability to immediately return after deformation expresses the elastic elongation ε_s_ and the A area proportional to the elastic energy dissipated. It characterizes the quality of the elastomer network capable of transferring high stresses. The delayed high-elastic elongation ε_hs_ and the B area proportional to the high-elastic energy dispersed characterizes the ability of the material to delayed recovery after deformation and the quality of the continuous phase capable of viscoelastic response. The permanent deformation ε_ps_ and the C area proportional to the energy accumulated correspond to the irreversible changes in the material, most likely related to the breakage of hard domain and disentanglement of polymer chains upon the initial stretching. This supposition confirms the occurrence of high-energy accumulation only in the first cycle.

It can be noticed that in the second and subsequent stretching cycles, all materials are characterized by elastic returns over 95% (in the fifth cycle it is already 100%). However, in the first cycle, terpolymers achieve elastic returns at only 70–80%. Most likely, this is due to the loss of energy for the spatial organization of the internal structure of the material. This behavior suggests that any products made of TPEEA or TPEAA must be pre-stretched (“trained”, mechanically conditioned) before their proper use.

**Figure 18:**
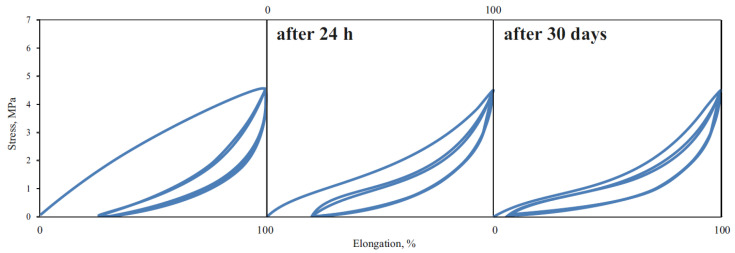
Mechanical hysteresis loops of the 3GT-DLAol-PA12 (polymer 12).

To confirm the proportionality of C area to energy absorbed to spatial orientation of domains (hard phase), the mechanical hysteresis loops of 24 h previously stretching material were made (Figure 18).

There was no large energy accumulation in the first cycle stretching in the previously stretched material. The area of the first loop is more than two times smaller than the corresponding area from the day before (C = 134; C_24h_ = 58) and is equal to the B area during the first stretch series (B = 59). This means that after 24 h, the domains do not return to the original location and the new spatial structure is thermodynamically stable.

The increase in the molar fraction of DLAol blocks results in better quality of the continuous phase. The volume of the polymer matrix (amorphous phase) increases, and hence crystalline domains are better dispersed. For this reason, dispersed energy (responsible for both elastic and highly elastic response) and accumulated energy in the first cycle decreases (an increase in the amorphous phase volume by 40% causes more than double the decrease of energy dispersed).

It is also worth noting that the percentage decrease in the elastic energy dispersed in the second and fifth cycles is relatively constant for each series and does not depend on the structure or volume of the amorphous phase (Table 8).

**Figure 19:**
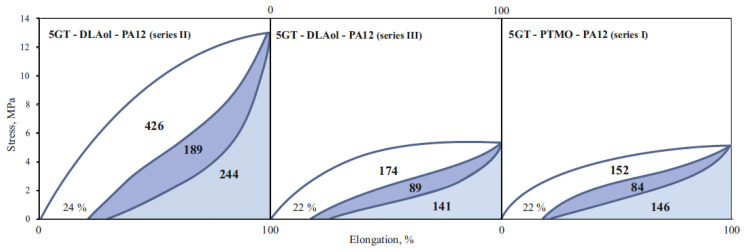
Mechanical hysteresis loops of the polymers 4, 9 and 14.

Figure 19 shows that TPEAAs are characterized by higher values of the energy accumulated in the first cycle than TPEEAs.

It can be assumed that terpolymers with PTMO matrix have a better elastomeric spatial network capable of transferring high stresses (more interfacial connections). The crystalline TPEEA domains are embedded in a matrix that allows them to move freely and thus return to the original position after releasing the tensile stress. Probably, the interactions at the domain–matrix interface, e.g., by entanglement of the chain, are relatively strong.

**Figure 20:**
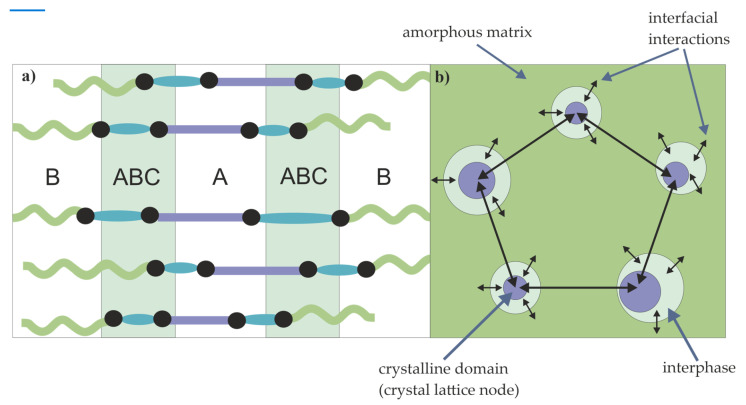
Predicted structure of TPEs; (**a**) phase composition: A—PA12, B—PTMO or DLAol, C—xGT; (**b**) physical structure.

Most likely, the domains move only within the interphase (area between the amorphous matrix and the crystalline domains composed of a mixture of PTT, PA12 and PTMO (or DLAol) blocks), which is anchored in the amorphous matrix (Figure 20). Thus, the more amorphous phase–interphase interfacial interactions occur, the greater the ability of the domains to return to a pre-stress position.

## 4. Conclusions

In the course of the research, new thermoplastic block elastomers were obtained. The synthesis of poly(ester-b-ether-b-amide) terpolymers consisted of two stages with the use of a titanium catalyst. The first step was the trans-esterification reaction of dimethyl terephthalate with glycol and the esterification reaction of α,ω-dicarboxylic oligo(laurolactam) with oligo(oxytetramethylene)diol or linoleic alcohol dimer, which was conducted simultaneously in different reactor. The next step was polycondensation reaction of the two previously obtained intermediate products.

To confirm the expected chemical structure of the terpolymers, ^13^C NMR, ^1^H NMR, and FT-IR methods were carried out. The FT-IR spectra contained peaks characteristic for ether, ester, amide, and aliphatic bonds. The absence of a broad peak in the range of 3300–3000 cm^−1^ for terpolymers, observed for dicarboxylic oligoamides, indicates that the block is incorporated into the macromolecule of the copolymer. This conclusion is supported by the occurrence of a peak at 39.56 ppm in the ^13^C NMR spectra.

In order to characterize the phase separation of the soft phase and the degree of crystallinity of the hard segment, a DSC analysis was performed. The method also allowed estimation of the size of the interphase and the presence of semicrystalline structures. The DMTA and WAXS techniques were used to confirm original conclusions. With a high degree of probability, the obtained copolymers are characterized by a crystalline-amorphous structure. The amorphous phases (matrix) consist of the flexible blocks PTMO or DLAol, contaminated by short ester chains. The crystalline domains are composed of the hard blocks PA12 and are infused by the xGT blocks. The increase in the number of carbons separating the terephthalate groups in the ester block slightly increases the amount of the amorphous phase in the TPE obtained.

The highest elastic residues were noted for terpolymers with 3 and 5 carbon atoms in their molecule. Terpolymers of series I exhibited generally better elastic properties, which was caused by the fact that flexibility due to ether bonds PTMO constituted the soft phase.

The obtained materials are characterized by a unique combination of properties such as low glass transition temperature, a wide temperature range of application, fast crystallization, good mechanical properties, including good elasticity, thermal stability and thermal and chemical resistance, and can find practical applications.

## Figures and Tables

**Table 1:** The composition of obtained copolymers.

Sample	Glycol	Soft Block	m_soft_	m_PA12_	m_DMT_	m_xG_	w_soft_	w_xGT_	w_PA12_	Series
1	2G	PTMO	3	1	5	9	0.520	0.133	0.347	I
2	3G	PTMO	3	1	5	9	0.515	0.141	0.343	
3	4G	PTMO	3	1	5	9	0.510	0.150	0.340	
4	5G	PTMO	3	1	5	9	0.505	0.158	0.337	
5	6G	PTMO	3	1	5	9	0.501	0.166	0.334	
6	2G	DLAol	3	1	5	9	0.369	0.175	0.456	II
7	3G	DLAol	3	1	5	9	0.365	0.185	0.450	
8	4G	DLAol	3	1	5	9	0.360	0.196	0.444	
9	5G	DLAol	3	1	5	9	0.356	0.204	0.440	
10	6G	DLAol	3	1	5	9	0.352	0.214	0.434	
11	2G	DLAol	5.5	1	5	9	0.518	0.134	0.349	III
12	3G	DLAol	5.5	1	5	9	0.513	0.142	0.345	
13	4G	DLAol	5.5	1	5	9	0.508	0.150	0.342	
14	5G	DLAol	5.5	1	5	9	0.504	0.157	0.339	
15	6G	DLAol	5.5	1	5	9	0.499	0.165	0.336	

2G—ethylene glycol; 3G—1,3-propanediol; 4G—1,4-butanediol; 5G—1,5-pentanediol; 6G—1,6-hexanediol; xGT—poly(multimethylene terephthalate) where x = 2–6; m—molar ratio; w—weight fraction.

**Table 2:** Wavenumbers and assignments of Fourier transform infrared (FT-IR) spectra band exhibited by obtained terpolymers.

3GT-PTMO-PA12 Band Frequency, cm^−1^	3GT-DLAol-PA12 Band Frequency, cm^−1^	Chemical Structure
3319	3294	Hydrogen-bonded N-H stretching and O-H stretching in the end groups
3081	3092	Fermi resonance of N-H,
2919 and 2850	2919 and 2849	CH_2_ asymmetric and symmetric stretching
1712	1717	Ester C=O stretching
1633	1635	Amide I, amide C=O stretching
1537	1544	Amide II, C-N stretching + amide C=O in plane bending
1465	1464	CH_2_ bending next to N-H group,
1408	1408	C=C stretching in the aromatic ring
1364	1374	O-H deformation and CH bending and CH_2_ twisting
1269	1267	Amide III, C-N stretching + amide C=O in plane bending
1204	-	O-H stretching
-	1162	CO-NH stretching
1099	1100	C-O-C asymmetric stretching
-	1061	C-O stretching in C-OH groups
1019	1018	C-H stretching in the aromatic ring
874	874	CH_2_ rocking in -O-(CH_2_)n-O- groups
727	729	Aromatic C-H bending out of plane
517	520	Amide IV

**Table 3:** Chemical shifts (ppm) of terpoly(ester-ether-amides) and terpoly(ester-aliphatic-amides).

3GT-PTMO-PA12 Band Frequency, ppm	3GT-DLAol-PA12 Band Frequency, ppm	Chemical Structure
218.5	218	C atoms of the amide group
172–173	172.6	C atoms of the carbonyl group
127–129	127	C atoms in the aromatic ring
78.6	-	C atoms in the ether group
61–65	62–66	CH2 groups linked to the carbonyl group through an oxygen atom
39.6	39.6	CH2 groups bonded to a carbonyl group
36.5	36.5	CH2 groups bonded to the C atom of the amide group
33	33	All other CH2 groups in the oligoamide chain
29.8	30	CH2 groups of β fragments bonded to amide or carbonyl groups or CH2 groups of β fragments of glycol
26.9	-	Polyether CH2 groups bonded to the other CH2 groups
-	20–26	C atoms in the aliphatic chain
-	14.4	C18 atoms in the aliphatic chain

**Table 4:** The properties of TPE with variable chemical structures of the ester block.

Polymer	[η], dL/g	H, Shore A	H, Shore D	p_H2O_, %	p_b_, %	Tm^i^, °C	Tm^e^, °C
1	1.25	84	29	2	134	135	150
2	1.40	76	26	2	174	112	128
3	1.69	76	24	4	182	139	151
4	1.30	78	24	3	174	128	135
5	1.32	75	23	2	204	146	150
6	1.30	84	19	1	84	133	148
7	1.55	87	19	1	82	125	138
8	1.74	87	19	1	75	141	164
9	1.36	69	16	0	96	129	150
10	1.25	69	16	4	117	150	171
11	1.29	81	16	1	140	130	148
12	1.53	73	15	0	171	114	120
13	1.68	75	15	0	179	142	154
14	1.31	71	13	1	180	120	147
15	1.29	71	13	2	199	142	162

[η]—limiting viscosity number; H—hardness; p_H2O_ and p_b_—absorbability of water and benzene, respectively; Tm^i^, Tm^e^—initial and end of melting point.

**Table 5:** Thermal properties of homopolymers and copolymers.

No.	Polymer	Tg_1_, °C	∆Cp_1_, J/g·°C	Tc_1_, °C	∆Hc_1_, J/g	Tm_1_ °C	∆Hm_1_, J/g	Tg_2_, °C	∆Cp_2_, J/g·°C	Tc_2_, °C	∆Hc_2_, J/g	Tm_2_, °C	∆Hm_2_, J/g
1	PTMO	−90	0.34	1	69.62	21	85.33	-	-	-	-	-	-
2	PTT	-	-	-	-	-	-	58	0.16	176	48.57	232	61.16
3	DLAol	−36	0.27	-	-	-	-	-	-	-	-	-	-
4	PA12	-	-	-	-	-	-	54	0.34	134	61.19	165	47.93
5	Vestamid L1700 [39]	-	-	-	-	-	-	37	0.17	151	77.9	179	81.0
6	PA12-PTMO [39]	−77	0.09	−3	23.5	20	21.0	-	-	134	37.0	163	39.5
7	PTT-PTMO [40]	−67	-	-	-	-	-	-	-	123	30.6	201	30.3
8	PA12-PTT	-	-	-	-	-	-	13	0.31	-	-	113	11.93

Tg_1_, Tm_1_, Tc_1_—glass transition, melting and crystallization temperatures of TPEEA in the low temperature region; ∆Cp_1_—specific heat capacity at Tg_1_; ∆Hm_1_—the enthalpy of melting at Tm_1_; ∆Hc_1_—the enthalpy of crystallization at Tc_1_; Tg_2_, Tm_2_, Tc_2_—glass transition, melting and crystallization temperatures of TPEEA in the high temperature region; ∆Cp_2_—specific heat capacity at Tg_2_; ∆Hc_2_—the enthalpy of crystallization at Tc_2_; ∆Hm_2_—the enthalpy of melting at Tm_2_.

**Table 6:** The properties of TPE with variable chemical structure of the ester block.

Polymer	δ_a_, MPa^1/2^	δ_c_, MPa^1/2^	δ*, MPa^1/2^
2GT	20.45	21.43	19.8–21.9 [41]
3GT	20.04	21.02	21–21.6 [42]
4GT	19.71	20.68	21.6–23.5 [43,44]
5GT	19.42	20.39	-
6GT	19.17	20.14	-
PTMO	17.65	18.60	17–18.6 [41]
PA12	23.05	24.35	22.1–23.8 [45,46,47]

δ_a_, δ_c_—solubility parameters theoretically calculated; δ*—solubility parameters empirically determined.

**Table 7:** Mechanical and elastic properties of terpolymers.

Sample	σ, MPa	ε, %	E, MPa	ε_ps_, %	ε_hs_, %	ε_s_, %	RII, %	RV, %	Series
1	6.9	112	18.1	24.2	1.1	75.5	98.9	99.7	I
2	4.7	213	13.6	24.6	1.4	75.4	98.6	99.8	
3	13.0	155	29.0	25.3	1.3	74.8	98.7	99.9	
4	6.5	301	21.1	22.2	1.2	77.5	98.8	100	
5	12.1	133	27.0	27.1	0.8	72.6	99.2	99.8	
6	17.1	99	25.4	29.0	2.8	71.1	97.2	99.6	II
7	14.5	198	23.6	29.1	2.7	70.7	97.4	99.5	
8	17.5	133	46.6	31.5	3.0	68.5	97.0	99.4	
9	14.4	183	29.8	28.3	2.5	71.4	97.5	99.4	
10	15.0	109	48.9	32.5	2.8	67.1	97.2	99.4	
11	6.0	98	15.2	24.4	1.9	76.1	98.3	99.5	III
12	2.6	202	13.1	23.1	1.5	75.1	99.5	100	
13	5.9	123	21.5	25.7	1.8	76.7	98.4	99.6	
14	4.0	149	9.1	22.2	1.7	77.8	98.9	100	
15	5.9	118	19.2	25.5	1.4	74.5	98.9	100	

σ—tensile strength; ε—elongation at break; E—Young’s modulus; ε_ps_—permanent elongation; ε_hs_—delayed high-elastic elongation; ε_s_—elastic elongation; RII and RV—elastic recovery in the second and fifth stretching cycle.

**Table 8:** Mechanical and elastic properties of terpolymers.

Sample	A	B_II_	B_V_	ΔB, %	C	Series
1	6.9	112	18.1	24.2	1.1	I
2	4.7	213	13.6	24.6	1.4	
3	13.0	155	29.0	25.3	1.3	
4	6.5	301	21.1	22.2	1.2	
5	12.1	133	27.0	27.1	0.8	
6	17.1	99	25.4	29.0	2.8	II
7	14.5	198	23.6	29.1	2.7	
8	17.5	133	46.6	31.5	3.0	
9	14.4	183	29.8	28.3	2.5	
10	15.0	109	48.9	32.5	2.8	
11	6.0	98	15.2	24.4	1.9	III
12	2.6	202	13.1	23.1	1.5	
13	5.9	123	21.5	25.7	1.8	
14	4.0	149	9.1	22.2	1.7	
15	5.9	118	19.2	25.5	1.4	

BII and BV—areas proportional to high-elastic energy dispersed during the second and fifth stretching cycle; ΔB—percentage change in the amount of high-elastic energy dispersed between the second and fifth cycles.

## Data Availability

The data presented in this study are available on request from the corresponding author.

## Appendix A

**Table A1:** Thermal properties of the TPEs of series I.

No.	Tg_1_, °C	∆Cp_1_, J/g·°C	Tc_1_, °C	∆Hc_1_, J/g	Tm_1_ °C	∆Hm_1_, J/g	Tm_2_, °C	∆Hm_2_, J/g	Tg_2_, °C	∆Cp_2_, J/g·°C	Tc_2_, °C	∆Hc_2_, J/g	Tm_3_, °C	∆Hm_3_, J/g	Tm_2_-Tg_1_
1	−72	0.18	-	-	−5	8	49	1.9	49	0.08	55	14.15	122	12.3	194
2	−73	0.28	-	-	−2	0.21	53	2.14	45	0.02	33	14.66	108	4.32	181
3	−72	0.16	-	-	−1	0.32	61	3.24	54	0.02	59	15.74	133	14.07	205
4	−73	0.29	-	-	4	5.15	53	1.37	57	0.03	60	13.23	127	11.63	200
5	−79	0.26	-	-	4	7.32	59	4.49	60	0.15	76	25.25	133	18.59	212

**Table A2:** Thermal properties of the TPEs of series II.

No.	Tg_1_, °C	∆Cp_1_, J/g·°C	Tc_1_, °C	∆Hc_1_, J/g	Tm_1_ °C	∆Hm_1_, J/g	Tm_2_, °C	∆Hm_2_, J/g	Tg_2_, °C	∆Cp_2_, J/g·°C	Tc_2_, °C	∆Hc_2_, J/g	Tm_3_, °C	∆Hm_3_, J/g	Tm_2_-Tg_1_
6	−21	0.12	-	-	-	-	57	3.67	-	-	44	14.56	122	13.93	143
7	−20	0.15	-	-	-	-	56	4.16	-	-	26	11.75	112	7.236	132
8	−21	0.13	-	-	-	-	55	2.84	-	-	53	18.40	130	17.40	151
9	−21	0.12	-	-	-	-	56	4.23	-	-	35	13.05	115	10.29	136
10	−23	0.17	-	-	-	-	56	3.35	-	-	53	17.57	129	15.67	152

**Table A3:** Thermal properties of the TPEs of series III.

No.	Tg_1_, °C	∆Cp_1_, J/g·°C	Tc_1_, °C	∆Hc_1_, J/g	Tm_1_ °C	∆Hm_1_, J/g	Tm_2_, °C	∆Hm_2_, J/g	Tg_2_, °C	∆Cp_2_, J/g·°C	Tc_2_, °C	∆Hc_2_, J/g	Tm_3_, °C	∆Hm_3_, J/g	Tm_2_-Tg_1_
11	−30	0.16	-	-	-	-	57	2.43	-	-	41	11.72	120	13.93	150
12	−32	0.15	-	-	-	-	56	3.11	-	-	31	6.52	113	3.22	145
13	−32	0.17	-	-	-	-	60	2.73	-	-	55	12.76	132	13.21	164
14	−31	0.18	-	-	-	-	57	3.71	-	-	37	11.98	125	11.02	156
15	−31	0.17	-	-	-	-	57	1.91	-	-	58	14.22	131	14.70	162
